# Establishment and comparison of three fear of progression risk prediction models for gynecological malignancies patients based on machine learning

**DOI:** 10.3389/fonc.2025.1632026

**Published:** 2025-10-21

**Authors:** Ao Xiong, JiaYi Wang, ZeNan Wang, DongYan Qi, YingQin Yu, Lei Xia, JunXiang Gao

**Affiliations:** ^1^ Office of Discipline Inspection and Supervision, The Second Hospital of Hebei Medical University, Shijiazhuang, Hebei, China; ^2^ The Graduate School of Hebei Medical University, Shijiazhuang, Hebei, China; ^3^ Department of Cardiology, The Second Hospital of Hebei Medical University, Shijiazhuang, Hebei, China; ^4^ Department of Radiotherapy, The Second Hospital of Hebei Medical University, Shijiazhuang, Hebei, China; ^5^ Department of Gynecology, The Second Hospital of Hebei Medical University, Shijiazhuang, Hebei, China

**Keywords:** gynecological malignancies, Fear of Progression, machine learning, prediction model, nursing

## Abstract

**Objective:**

This study applied the Society Ecosystems Theory to investigate Fear of Progression (FoP) prevalence and predictors in gynecological malignancy patients. By constructing and comparing three machine learning models, we sought to identify the optimal scientifically validated predictive tool for FoP risk in clinical practice, thereby enabling early identification of high-risk populations and informing evidence-based targeted interventions.

**Methods:**

A convenience sample of 330 patients diagnosed with gynecological malignancies was recruited from a tertiary hospital in China between September 2023 and August 2024. Data were collected through validated instruments: the General Information Questionnaire, Fear of Progression Questionnaire-Short Form, Comprehensive Scores for Financial Toxicity, Chinese Dyadic Coping Inventory, Perceived Social Support Scale, and Chinese Memorial Symptom Assessment Scale. The dataset was partitioned into training (70%, *n* = 231) and testing sets (30%, *n* = 99) using stratified random sampling. Patients were classified into FoP and non-FoP groups based on diagnostic criteria. Three machine learning algorithms, logistic regression (LR), support vector machine (SVM), and random forest (RF) were implemented to develop FoP prediction models. Model performance was compared using accuracy, recall, precision, F1-score, and area under the ROC curve (AUC-ROC) to select the optimal model.

**Results:**

This study included 330 patients with gynecological malignancies, with a FoP incidence of 52.7% (n = 174). All three models identified social support, dyadic coping, mindset bias, and elevated tumor markers as significant predictors of FoP (*P<* 0.05). Additionally, symptom distress and financial toxicity demonstrated significant predictive value in the SVM and RF models. Comparative analysis revealed that the RF model outperformed the LR and SVM models in overall predictive performance.

**Conclusions:**

The Random Forest-based prediction model exhibited optimal performance, demonstrating high accuracy and reliability in identifying FoP risk among gynecological malignancy patients. It can provide a scientific foundation for early FoP detection and personalized intervention strategies. These findings underscore the clinical utility of combining machine learning approaches with social-ecological theory to advance precision nursing practices in psycho-oncology care.

## 1 Introduction

Globally, gynecological malignancies pose a severe threat to women’s health, accounting for 15%–20% of female cancer cases (1). In 2022, approximately 1.48 million new cases and 670,000 deaths were reported worldwide, with China alone recording 290,000 new diagnoses and 100,000 deaths—a trend marked by increasing incidence among younger populations (2). Although advances in diagnosis and treatment have significantly improved survival rates, patients continue to endure dual physiological and psychological burdens, with their quality of life compromised by treatment side effects, financial strain, and fear of recurrence (3). Fear of Progression (FoP), recognized as one of the most prevalent unmet needs in cancer patients (4), manifests as excessive distress about disease deterioration or relapse, accompanied by adverse effects on physical, psychological, and social functioning (5). Studies indicate that 20%–70% of patients experience clinically significant FoP (6). While moderate FoP may enhance health vigilance and self-management, excessive FoP can precipitate depression, anxiety, post-traumatic stress, reduced treatment adherence, and impaired social adaptation (7).

FoP is a common psychological response in cancer patients whose development and persistence do not stem from isolated causes. Patients with gynecological malignancies not only endure the physical and psychological burdens of the disease but also face significant financial toxicity. Research indicates a positive correlation between symptom distress and FoP levels in cancer patients (8). The substantial costs of anticancer therapies and associated financial pressures not only impose objective economic burdens but also evoke subjective distress, such as anxiety and perceived helplessness, further exacerbating FoP severity (9). By providing positive dyadic coping and emotional support, supportive dyadic relationships during illness adaptation play a crucial role in alleviating patients’ anxiety and reducing FoP levels (10). Moreover, multidimensional social support from family, friends, and healthcare professionals has been empirically shown to enhance patients’ ability to manage uncertainty, mitigate psychological stress, and consequently diminish FoP (11).

The Society Ecosystems Theory (SET) posits that patients’ physical and mental health are susceptible to multifactorial influences across the microsystem (encompassing intrapersonal and biological mechanisms), mesosystem (reflecting interpersonal and familial interactions), and macrosystem (involving broader sociocultural and institutional structures) (12). This theoretical framework aims to elucidate the complex and dynamic interactions between human behavior and social environments (13). Within this context, FoP levels are shaped not only by individual psychological and physiological states but also by familial environments (e.g., couples’ stress-coping capacities, financial status) and the extent of social support. The interplay and reinforcement across these levels are pivotal in facilitating patients’ disease adaptation. Thus, integrating multilevel influencing factors based on SET provides a foundational framework for precise FoP identification and prediction.

Risk prediction is pivotal for identifying high-FoP populations (14). Current FoP studies predominantly rely on traditional logistic regression (15, 16), underutilizing the technical advantages of machine learning (ML). With the rise of precision medicine, ML has emerged as a cornerstone technology for medical prediction due to its robust data-mining capabilities (17). Among ML algorithms, logistic regression (LR) excels in risk factor identification (18), support vector machines (SVM) demonstrate stability with small samples (19), and random forests (RF) efficiently handle high-dimensional data (20). Yet, few studies have developed FoP prediction models for gynecological malignancies through multi-algorithm comparisons.

Guided by the Society Ecosystems Theory, this study constructs FoP risk prediction models using ML. Data were collected via self-designed and standardized scales, encompassing multidimensional variables such as demographic characteristics, symptom distress, financial toxicity, dyadic coping, and social support. By comparing the performance of LR, SVM, and RF algorithms, this study aims to: (1) establish an integrated biopsychosocial predictive tool; and (2) identify key predictors to inform tiered psychological interventions. The findings will facilitate early clinical identification of high-risk patients and optimize the allocation of mental health resources.

## 2 Methods

### 2.1 Study design

This cross-sectional study utilized a convenience sampling method to recruit patients with gynecological malignancies hospitalized at a tertiary hospital.

### 2.2 Setting

Participants were recruited from the gynecology, radiotherapy, and oncology departments of the same tertiary hospital between September 2023 and August 2024.

### 2.3 Participants

A total of 342 patients were initially participated through convenience sampling. Inclusion criteria comprised: (1) pathologically confirmed diagnosis of gynecological malignancies (including uterine corpus, cervical, ovarian, fallopian tube, vaginal, and vulvar cancers); (2) diagnosis duration ≥1 month; (3) age ≥18 years with intact cognitive and communication abilities; (4) awareness of diagnosis and voluntary participation. Exclusion criteria included: (1) absence of a spouse; (2) comorbid psychiatric or cognitive disorders; (3) concurrent non-gynecological malignancies; (4) severe cardiopulmonary, hepatic, renal, or other systemic comorbidities. Sample size calculation followed the events/variable method (21), requiring 10–20 participants per predictor variable. With 20 independent variables and considering the 10% shedding rate, the minimum sample size should be 220. After excluding 12 invalid responses (e.g., incomplete questionnaires), 330 patients were included in the final analysis.

### 2.4 Ethical considerations

This study received ethical approval from the Ethics Committee of the hospital (Approval No.: 2023-R557) prior to data collection. Research team members consulted nursing staff in relevant departments to confirm patient eligibility. Meanwhile, the researchers explained the purpose and significance of the study to eligible patients and their families. Patients who agreed to participate provided written informed consent and subsequently completed the questionnaire by scanning a QR code.

### 2.5 Data collection

Data were collected by two trained nursing postgraduate students proficient in standardized scale administration. The study utilized the Wenjuanxing platform (a widely used online survey tool in China) for face-to-face questionnaire administration. Researchers provided uniform explanations of the questionnaires’ objectives, confidentiality protocols, and completion guidelines to patients and families. After obtaining informed consent, participants received QR codes to access electronic questionnaires. Patients independently completed the surveys based on their personal circumstances, with researchers available to clarify any ambiguities in real time. For participants unable to self-administer the survey, a structured interview format was implemented: researchers read items aloud using neutral language, recorded verbal responses, and objectively transcribed answers. Electronic submissions were systematically reviewed, excluding questionnaires with short completion times (<3 minutes) or patterned responses.

### 2.6 Survey instruments

Data were collected through self-administered questionnaires encompassing 20 variables across individual, familial, and societal dimensions.

#### 2.6.1 General information questionnaire

This instrument captured demographic characteristics (age, employment status, residence, number of children, education level, health insurance type, monthly household income per capita, mindset bias) and clinical parameters (family history of cancer, cancer type, treatment modalities, time since diagnosis, comorbidities, cancer stage, tumor marker elevation, and HPV infection).

#### 2.6.2 Chinese memorial symptom assessment scale

The MSAS-Ch evaluates symptom experiences over the preceding seven days (22). This 32-item scale comprises four subscales: physical symptoms, psychological symptoms, global distress index, and total MSAS score. Twenty-four items assess symptom prevalence, frequency, severity, and distress using 4-point Likert scales, while eight items measure frequency and severity only. Distress levels are rated on a 5-point Likert scale, with higher scores indicating greater symptom distress. The scale demonstrated good reliability (Cronbach’s α: 0.79–0.87).

#### 2.6.3 Comprehensive score for financial toxicity

The 12-item COST-PROM assesses financial toxicity using 5-point Likert scales (excluding item 12 from scoring). Total scores range from 0 to 44, with lower values indicating severe financial toxicity (23). Validation studies reported excellent internal consistency (Cronbach’s α: 0.889).

#### 2.6.4 Chinese version of the dyadic coping inventory

This 37-item instrument evaluates five dimensions of dyadic coping: stress communication, supportive coping, delegated coping, negative coping, and common coping. Responses are recorded on a 5-point Likert scale (1 = “rarely” to 5 = “very frequently”), with total scores ranging from 35 to 175. Higher scores reflect more frequent mutual supportive behaviors between couples (24). The scale demonstrated strong reliability (Cronbach’s α: 0.84).

#### 2.6.5 Perceived social support scale

The PSSS measures social support across three domains (family, friends, others) using 12 items rated on a 7-point Likert scale (1 = “strongly disagree” to 7 = “strongly agree”). Total scores range from 12 to 84, with higher values indicating stronger perceived support (25). The Chinese version showed good reliability (Cronbach’s α: 0.84).

### 2.7 Outcome measure

Fear of Progression Questionnaire-Short Form (FoP-Q-SF). This 12-item scale assesses FoP across physical health and social/family functioning domains. Patients rate items on a 5-point Likert scale (1 = “never” to 5 = “always”), with total scores ranging from 12 to 60. A clinical cutoff of ≥34 identifies significant FoP severity, where higher scores indicate greater progression-related fears (26). The instrument demonstrated strong internal consistency (Cronbach’s α: 0.883).

### 2.8 Statistical analysis

Data analysis and modeling were performed using SPSS 25.0 and R studio 4.4.0. Normally distributed continuous variables were expressed as mean ± standard deviation ( $\bar{x}$ ± s) and compared using Student’s t-test, while non-normally distributed continuous variables were reported as median (interquartile range) and analyzed via the Mann-Whitney U test. Categorical variables were summarized as frequencies (%) and compared using chi-square tests. Statistical significance was set at *P* < 0.05 (two-tailed). For predictive modeling, the dataset was randomly partitioned into training (70%) and testing (30%) sets. Three machine learning algorithms were implemented: LASSO-regularized logistic regression, support vector machine, and random forest. Model performance was evaluated using receiver operating characteristic (ROC) curves, area under the curve (AUC), accuracy, recall, precision, and F1-score.

## 3 Results

### 3.1 Prevalence of FoP in gynecological malignancies patients

This study enrolled 330 patients with gynecological malignancies. The mean FoP score was 34.62 ± 9.29 (range: 12–60), with 174 patients (52.7%) exceeding the clinical cutoff score (≥34) for significant FoP. The subgroup with FoP demonstrated a mean score of 42.36 ± 4.90. The cohort was randomly divided into a training set (70%, n = 221) and a testing set (30%, n = 99). Baseline characteristics showed no statistically significant differences between the training and testing sets (*P* > 0.05). Furthermore, the prevalence of FoP did not differ significantly between the two sets (*χ²* = 1.565, *P* = 0.211), confirming balanced distribution of outcome variables and covariates across the partitioned datasets.

### 3.2 Univariate analysis of factors associated with FoP in gynecological malignancies patients

The training set was stratified into a non-FoP group (*n* = 156) and FoP group (*n* = 174) based on clinical FoP status (cutoff ≥ 34). Univariate analysis of sociodemographic and clinical variables revealed statistically significant differences (*P* < 0.05) between groups across 16 predictors: age, employment status, residence, education level, health insurance type, monthly household income per capita, family history of cancer, cancer type, time since diagnosis, comorbidities, mindset bias, elevated tumor markers, financial toxicity, dyadic coping, social support, and symptom distress (**Table 1**). These findings preliminarily identified candidate predictors spanning biological, psychological, and socioeconomic domains for subsequent multivariate modeling.

**Table 1:** Univariate analysis of factors associated with FoP in gynecological malignancies patients (n=330).

Variables	Non-FoP(n=156)	FoP(n=174)	z/χ^2^	*P*-value
Age,[year, n (%)]				
<45	16 (10.3)	51 (29.3)	35.542	<0.001
45~59	53 (34)	78 (44.8)		
>59	87 (55.8)	45 (25.9)		
Employment status,n (%)				
Employed or medical leave	19 (12.2)	67 (38.5)	56.411	<0.001
Retired	55 (35.3)	11 (6.3)		
Unemployed	82 (52.6)	96 (55.2)		
Residence,n (%)				
Rural	75 (48.1)	115 (66.1)	10.929	0.001
Urban	81 (51.9)	59 (33.9)		
Number of children, n (%)				
0	1 (0.6)	5 (2.9)	5.683	0.128
1	39 (25)	57 (32.8)		
2	78 (50)	79 (45.4)		
≥3	38 (24.4)	33 (19)		
Education level, n (%)				
Junior high school or below	81 (51.9)	109 (62.6)	6.483	0.039
High school or vocational secondary	46 (29.5)	31 (17.8)		
College or above	29 (18.6)	34 (19.5)		
Health insurance type, n (%)				
Employee medical insurance	41(26.3)	30(17.2)	32.326	<0.001
Resident basic medical insurance	85(54.5)	116(66.7)		
Self-payment	1(0.6)	19(10.9)		
Commercial insurance + employee/resident	29(18.6)	9(5.2)		
Monthly household income per capita[RMB, n (%)]				
<2000	15 (9.6)	57 (32.8)	78.173	<0.001
2000~<5000	27 (17.3)	73 (42)		
5000~<8000	61 (39.1)	30 (17.2)		
≥8000	53 (34)	14 (8)		
Family history of cancer, n (%)				
Yes	16 (10.3)	44 (25.3)	12.492	<0.001
No	140 (89.7)	130 (74.7)		
Cancer type, n (%)				
Cervical cancer	57(36.5)	76(43.7)	13.128	0.011
Ovarian cancer	56(35.9)	58(33.3)		
Endometrial cancer	28(17.9)	33(19)		
Other types	15(9.6)	4(2.3)		
Mixed types	0(0)	3(1.7)		
Treatment modalities, n (%)				
Surgery/Radiotherapy	3(1.9)	2(1.1)	0.381	0.944
Surgery+Chemotherapy/Radiotherapy	85(54.5)	96(55.2)		
Chemotherapy+Radiotherapy	19(12.2)	20(11.5)		
Surgery+Radiotherapy+Chemotherapy	49(31.4)	56(32.2)		
Time since diagnosis[month, n (%)]				
1~<4	76 (48.7)	135 (77.6)	31.708	<0.001
4~<7	46 (29.5)	18 (10.3)		
7~<12	9 (5.8)	8 (4.6)		
≥12	25 (16)	13 (7.5)		
Comorbidities, n (%)				
Yes	41(26.3)	69 (39.7)	6.62	0.01
No	115 (73.7)	105 (60.3)		
Mindset bias, n (%)				
Positive	114 (73.1)	20 (11.5)	139.011	<0.001
Neutral	31 (19.9)	63 (36.2)		
Negative	11 (7.1)	91 (52.3)		
Cancer stage, n (%)				
I	59 (37.8)	47 (27)	6.362	0.095
II	32 (20.5)	35 (20.1)		
III	49 (31.4)	76 (43.7)		
IV	16 (10.3)	16 (9.2)		
Tumor marker elevation, n (%)				
Yes	70 (44.9)	140 (80.5)	45.019	<0.001
No	86 (55.1)	34 (19.5)		
HPV infection status, n (%)				
Yes	64 (41)	90 (51.7)	3.783	0.052
No	92(59)	84(48.3)		
Financial toxicity[score, M (P_25_, P_75_)]	36(27,39)	9(5,18)	-11.202	<0.001
Dyadic coping[score, M (P_25_, P_75_)]	151(140,158)	89.5(64,106.25)	-11.012	<0.001
Social support[score, M (P_25_, P_75_)]	72(67.25,76)	44(32,50)	-12.056	<0.001
Symptom distress[score, M (P_25_, P_75_)]	1.56(1.28,2)	2.75(2.5,3.32)	-11.684	<0.001

### 3.3 Prediction models for FoP in gynecological malignancies patients

#### 3.3.1 Logistic regression model

**Figure 1:**
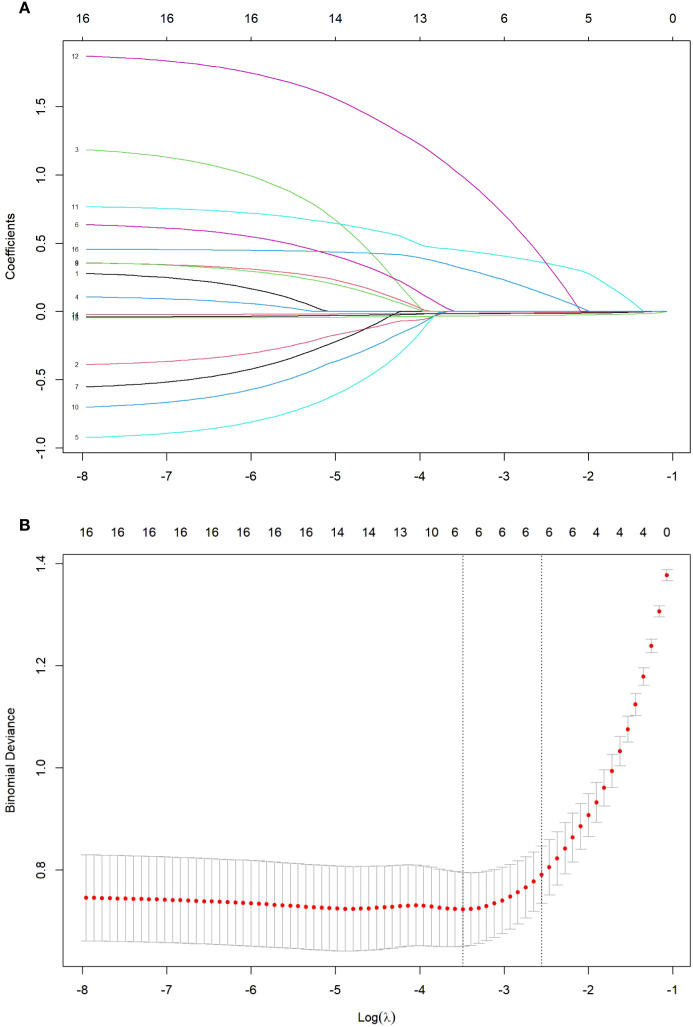
**(A)** LASSO regression coefficient path plot. **(B)** Cross-validation plot of the optimal parameter λ.

Using FoP occurrence as the dependent variable, variables with statistically significant differences identified in the univariate analysis (**Table 1**) were incorporated into LASSO regression, with coding schemes detailed in **Table 2**. As illustrated in **Figure 1**, panel (A) displays the coefficient trajectories of each variable during regularization, while panel (B) presents the cross-validated mean squared error (MSE) curve, where the vertical dashed lines denote the optimal *λ* values (lambda.min and lambda.1se). The lambda.min (*λ* = 0.03053) was selected as the optimal regularization parameter, yielding six non-zero coefficients: social support (*β* = -0.0326), financial toxicity (*β* = -0.0158), dyadic coping (*β* = -0.0121), symptom distress (*β* = 0.2854), negative mindset bias (*β* = 0.4334), and elevated tumor markers (*β* = 0.8896).

**Table 2:** Variable coding scheme.

Variable	Coding scheme
Fear of Progression	Non-FoP=0, FoP=1
Age	<45=1, 45~59=2, >59=3
Employment status	Employed or medical leave=1, Retired=2,Unemployed=3
Residence	Rural=1,Urban=2
Education level	Junior high or below=1, High school or vocational secondary=2, College or above=3
Family cancer history	Yes=1, No=0
Health insurance type	Employee insurance=1, Resident insurance=2, Self-payment=3, Commercial insurance + employee/resident=4
Monthly Household Income per capita	<2000=1, 2000~<5000=2, 5000~<8000=3, ≥8000=4
Cancer type	Cervical cancer=1, Ovarian cancer=2, Endometrial cancer=3, Other types=4, Mixed types=5
Time since diagnosis	1~<4=1, 4~<7=2, 7~<12=3, ≥12=4
Comorbidities	Yes=1, No=0
Mindset bias	Positive=1, Neutral=2, Negative=3
Tumor marker elevation	Yes=1, No=0
Financial toxicity	Continuous variable (raw score)
Dyadic coping	Continuous variable (raw score)
Social support	Continuous variable (raw score)
Symptom distress	Continuous variable (raw score)

Using the occurrence of FoP as the dependent variable (non-FoP = 0, FoP = 1), six factors identified as statistically significant (*P<* 0.05) in LASSO regression were incorporated as independent variables in the logistic regression analysis, with variable coding consistent with **Table 2**. The results demonstrated that neutral mindset bias (*OR* = 2.494, *P* = 0.038), negative mindset bias (*OR* = 3.563, *P* = 0.026), and elevated tumor markers (*OR* = 4.727, *P* < 0.001) emerged as significant risk factors for FoP in patients with gynecological malignancies. Conversely, dyadic coping (*OR* = 0.985, *P* = 0.035) and social support (*OR* = 0.962, *P* = 0.020) were identified as protective factors against FoP (**Table 3**).

**Table 3:** Results of logistic regression analysis for factors associated with FoP in patients with gynecological malignancies (n=330).

Variable	Regression coefficient (*β*)	Standard error (SE)	Wald*χ^2^*	*P*-value	Odds ratio (*OR*)	95% Confidence interval(95% CI)
Constant	1.141	1.500	0.579	0.447	3.130	—
Mindset bias (Neutral)	0.914	0.439	4.323	0.038	2.494	[1.054~5.901]
Mindset bias (Negative)	1.270	0.572	4.936	0.026	3.563	[1.162~10.927]
Elevated tumor markers (Yes)	1.553	0.367	17.878	<0 .001	4.727	[2.301~9.712]
Dyadic coping	-0.015	0.007	4.436	0.035	0.985	[0.971~0.999]
Social support	-0.039	0.017	5.412	0.020	0.962	[0.931~0.994]

**Figure 2:**
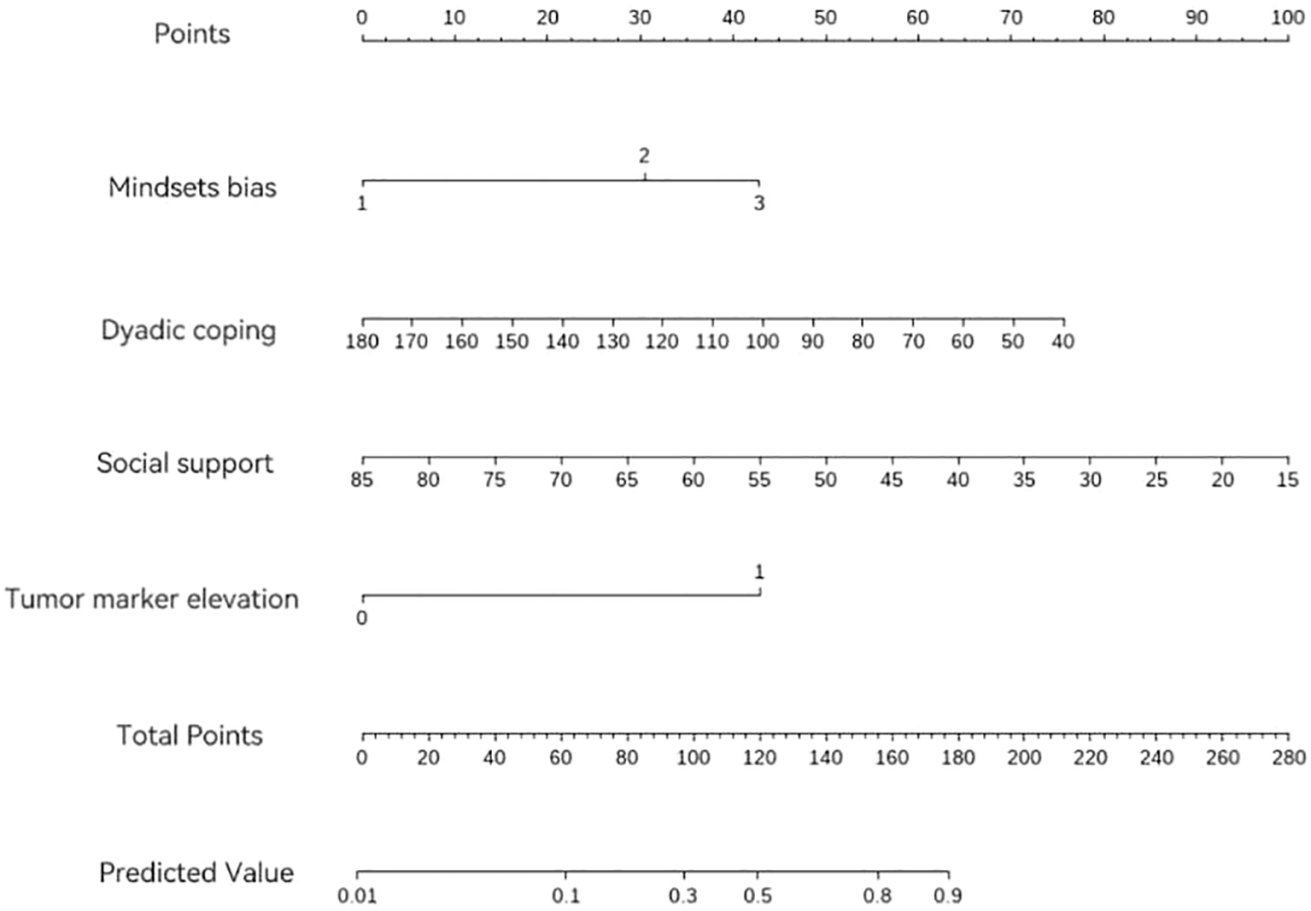
Nomogram for predicting FoP in patients with gynecological mMalignancies.

The results were further visualized through a nomogram to enhance clinical interpretability, as detailed in **Figure 2**.

#### 3.3.2 Support vector machine model

**Figure 3:**
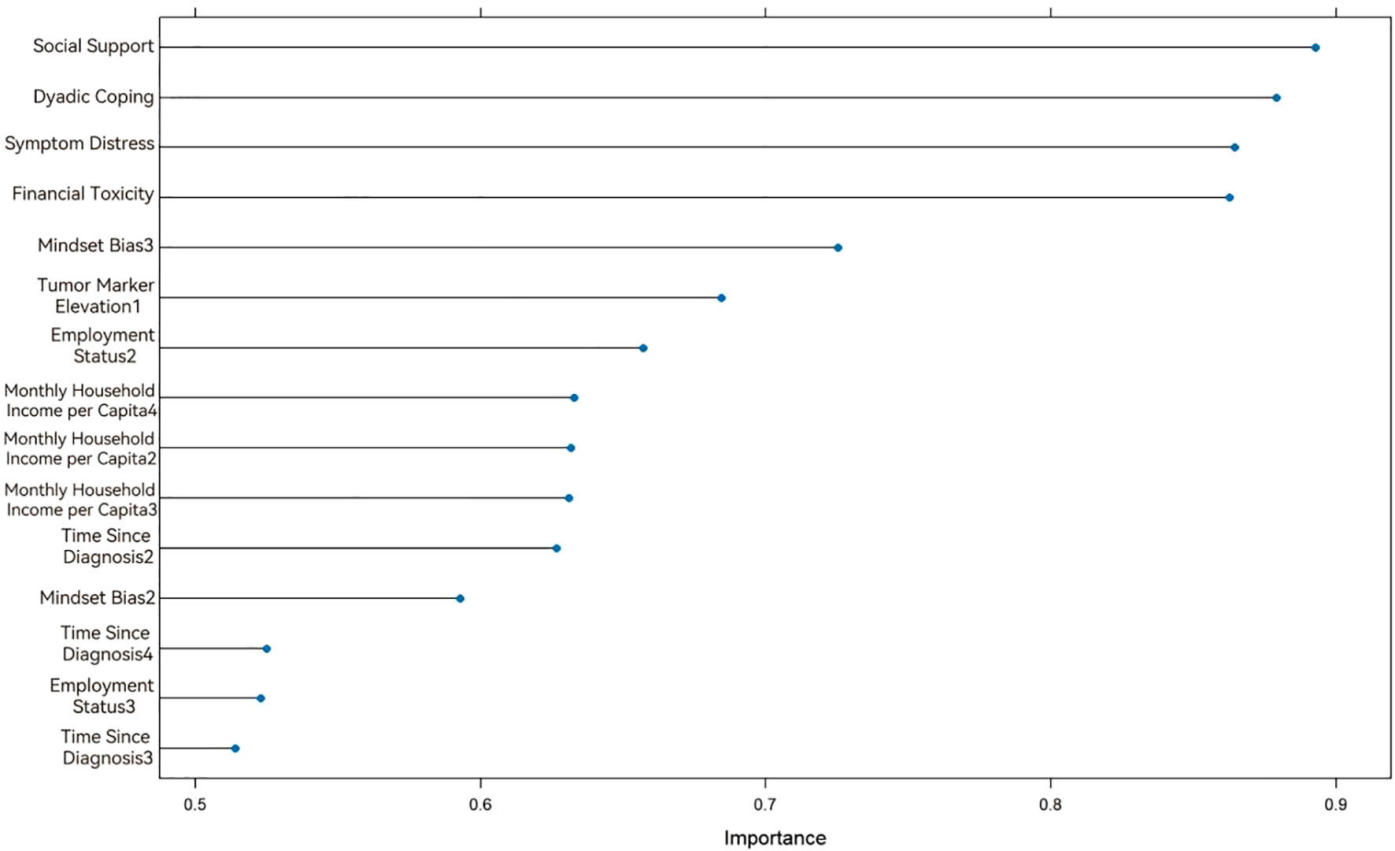
Variable importance ranking in the Support Vector Machine model.

The SVM model was developed using a radial basis function (RBF) kernel. To enhance model performance, Bayesian hyperparameter optimization integrated with cross-validation was applied to refine the penalty parameter *C* and kernel parameter *γ*. The optimal hyperparameters were identified as *C* = 0.827 and *γ* = 0.0117. Subsequently, recursive feature elimination (RFE) was implemented, yielding nine variables with significant predictive influence. **Figure 3** illustrates the ranked importance of these selected variables.

#### 3.3.3 Random forest model

**Figure 4:**
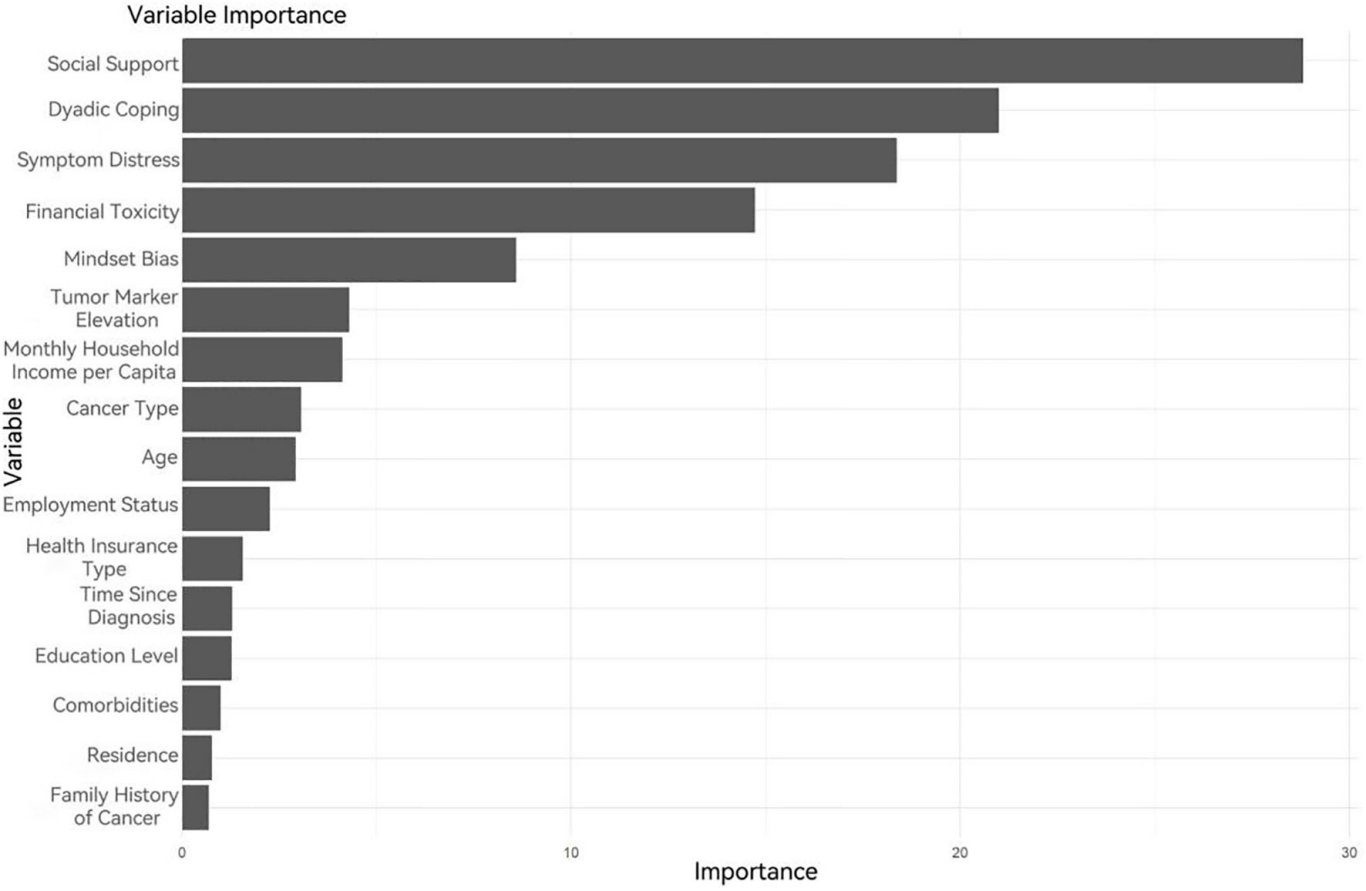
Variable importance ranking in the Random Forest model.

Random Forest, as a robust machine learning model, demonstrates performance highly dependent on the configuration of its hyperparameters. To develop an optimal predictive model, Bayesian hyperparameter optimization was employed to identify optimal configurations for critical parameters: the number of randomly selected features per node (mtry = 2), the number of decision trees (ntree = 62), the maximum tree depth (max_depth = 3), and the minimum node size (min_node_size = 49). The results of this optimization process are visualized in **Figure 4**.

### 3.4 Common predictive factors

In this study, three distinct machine learning models were developed to predict FoP in patients with gynecological malignancies, enabling systematic screening and analysis of associated factors. The results revealed that social support, dyadic coping, mindset bias, and elevated tumor markers were consistently identified as shared predictors across all three models. Furthermore, symptom distress and financial toxicity ranked prominently in variable importance analyses for both the SVM and RF models, thereby establishing their significance as critical predictors of FoP in this population. These variables collectively encompass individual, familial, and societal dimensions, further validating the feasibility of the Society Ecosystems Theory as the study’s conceptual framework. This theory emphasizes the dynamic interplay between individuals, families, and broader societal contexts, underscoring the relevance of multilevel factors in predicting psychological states and disease trajectories. The findings demonstrate the adaptability and efficacy of the theoretical model in addressing real-world clinical challenges, providing multidimensional support for FoP prediction and establishing a theoretical foundation for psychosocial interventions and management in gynecological oncology care.

### 3.5 Comparative performance of the three prediction models

In the training set, all three models achieved accuracy, recall, precision, and F1-scores exceeding 0.85. The RF model demonstrated superior performance, with accuracy = 0.918, recall = 0.894, precision = 0.925, and F1-score = 0.910, outperforming both logistic regression (LR) and SVM models. The area under the ROC curve (AUC) values ranked as follows: RF model (0.968) > LR model (0.933) > SVM model (0.920). In the testing set, while the SVM model exhibited a lower recall (0.792), all other metrics for the three models remained above 0.80. The RF model again showed optimal performance, achieving accuracy = 0.900, recall = 0.885, and F1-score = 0.889, though its precision (0.893) was slightly lower than that of the LR model (0.905). AUC values in the testing set followed the same hierarchy: RF model (0.928) > LR model (0.898) > SVM model (0.889).

**Figure 5:**
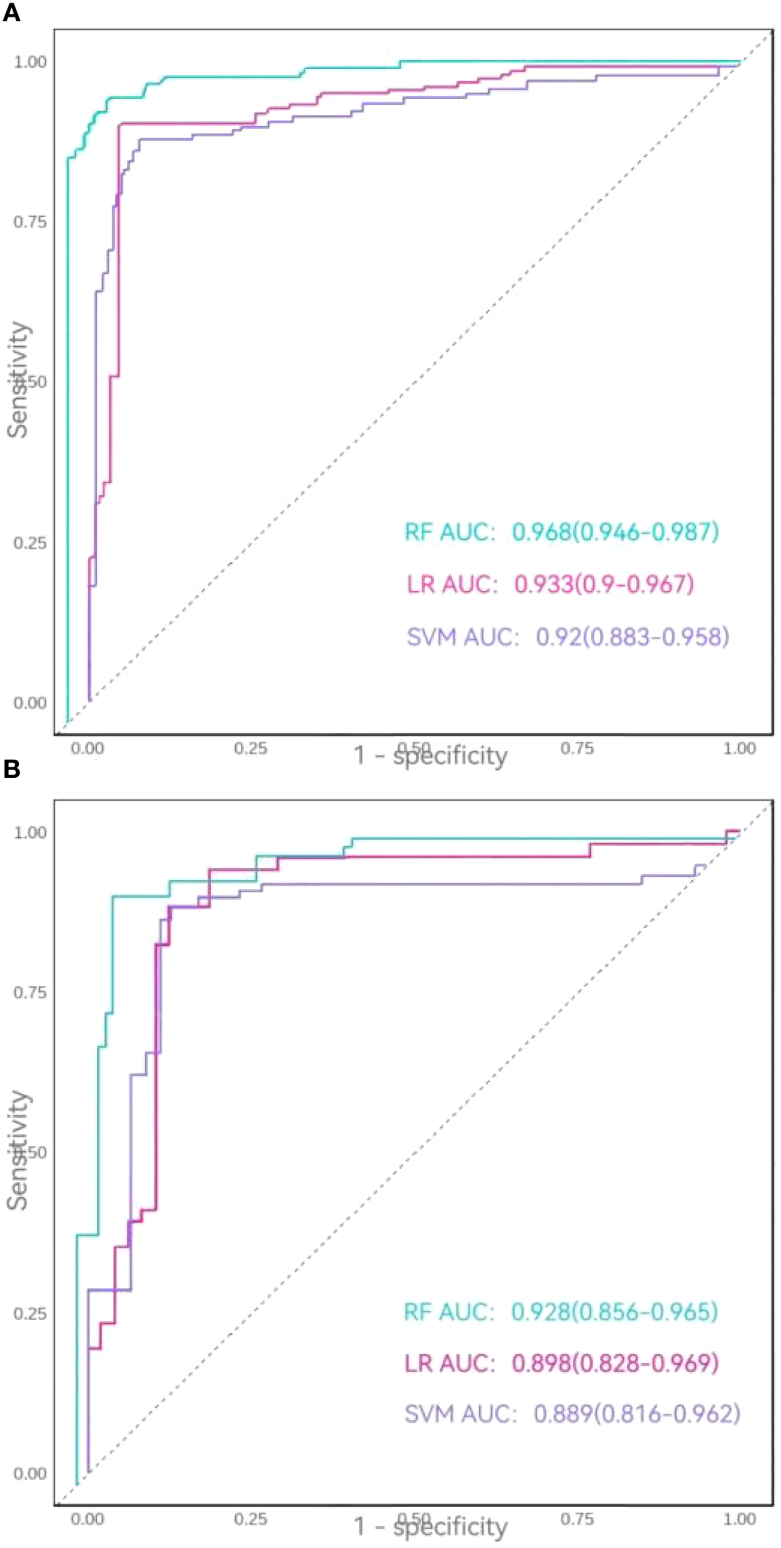
**(A)** ROC curves of the three models in the training set. **(B)** ROC curves of the three models in the testing set.

Thus, comparative analysis confirmed the Random Forest model as the most robust predictor, demonstrating superior overall performance relative to both logistic regression and support vector machine models (**Table 4**, **Figures 5A, B**).

**Table 4:** Comparative performance of the three prediction models.

Dataset	Method	Accuracy	Recall	Precision	F1-score	AUC
Training Set	Logistic Regression	0.896	0.875	0.907	0.891	0.933
	Support Vector Machine	0.887	0.889	0.873	0.881	0.920
	Random Forest	0.918	0.894	0.925	0.910	0.968
Testing Set	Logistic Regression	0.859	0.843	0.905	0.874	0.898
	Support Vector Machine	0.859	0.792	0.886	0.839	0.889
	Random Forest	0.900	0.885	0.893	0.889	0.928

## 4 Discussion

### 4.1 Current status of FoP in gynecological malignancies patients

This study identified a FoP prevalence of 52.7% among patients with gynecological malignancies, consistent with findings by Su et al. (27) in Chinese gynecological cancer patients (56%) but higher than the rate reported by Ye et al. (28) in patients with malignant bone tumors (45.4%). This discrepancy underscores both the pervasiveness of FoP in gynecological oncology populations and its critical relevance to clinical care. The elevated prevalence may be attributed to the following interrelated factors. First, societal role transitions and familial responsibility realignment play a pivotal role. As a predominantly female population, gynecological malignancy patients often shoulder substantial caregiving roles within families. The onset of illness alters patients’ societal roles, potentially diminishing their self-worth while perpetuating anxieties about becoming a familial burden. Such role conflicts compound psychological distress and amplify disease-related fears. Second, body image and reproductive function impairment exert profound psychological impacts. Treatments for gynecological malignancies, particularly surgeries involving reproductive organ resection, not only disrupt physiological functions but also profoundly affect body integrity, gender identity, intimate relationships, and fertility. Postoperative physical changes—such as scarring or organ loss—may erode self-confidence and challenge gender perception. For younger patients, fertility loss constitutes a particularly devastating consequence, with many experiencing profound shifts in bodily identity and self-perception post-surgery (3). Concurrently, strained marital relationships and intimacy challenges may further exacerbate psychological burdens, collectively intensifying FoP severity. Third, disease progression patterns and treatment prognosis contribute substantially to sustained fears. Despite advancements in therapeutic interventions that have improved survival rates, the latent risks of progression, recurrence, and metastasis persist as significant psychological stressors. Certain subtypes, such as ovarian cancer, exhibit insidious progression coupled with limited sensitivity of biomarkers for recurrence monitoring, trapping patients in chronic hypervigilance and psychological exhaustion. Furthermore, treatment modalities like chemotherapy and radiotherapy often induce prolonged physical discomfort (e.g., fatigue, nausea), amplifying health-related uncertainties. These findings highlight the imperative for precision-driven predictive tools to enable early identification of high FoP risk in this population, thereby facilitating timely psychosocial interventions.

### 4.2 Predictive factors for FoP in gynecological malignancy patients

The three models consistently identified social support, dyadic coping, mindset bias, and elevated tumor markers as shared predictors of FoP. Symptom distress and financial toxicity ranked prominently in variable importance analyses for both SVM and RF models, suggesting their potential significance as critical predictors. Consequently, these six factors were collectively analyzed as predictors of FoP in this population.

#### 4.2.1 Social support

This study revealed social support as a core protective factor against FoP in gynecological malignancy patients. In the logistic regression model, social support demonstrated an *OR* = 0.962,*P* = 0.020, while it emerged as the top-ranking predictor in both SVM and RF models. Hu et al. (7) corroborated these findings, reporting that patients with low social support levels exhibited heightened fear of disease progression, further validating the universal role of social support in mitigating psychological distress among cancer populations. Research indicates that social support alleviates stress responses by providing emotional and informational resources, enabling patients to better cope with cancer-related stressors, thereby reducing negative emotional states and enhancing quality of life (29). Low social support is strongly associated with elevated FoP levels. When confronting gynecological malignancies, patients lacking effective support from family, friends, or healthcare systems often experience profound isolation, exacerbating disease-related fears. Conversely, robust social support networks serve as psychological buffers, alleviating emotional burdens and fostering resilience. This not only mitigates negative affect but also improves treatment adherence and rehabilitation engagement, ultimately reducing FoP risk. Notably, many patients in this cohort remained in the stress adaptation phase following cancer diagnosis, characterized by overwhelming psychological pressure and fear of the unknown—emotions frequently surpassing their coping capacities. While patients may actively seek external support through sharing concerns, such efforts may inadequately address entrenched fears in certain contexts. This underscores the necessity for targeted social support interventions, particularly during critical peri-treatment periods. Beyond emotional and informational aid, clinicians should encourage patients to rebuild social networks through structured activities, thereby enhancing perceived social support efficacy.

#### 4.2.2 Dyadic coping

This study identified dyadic coping as a protective factor against FoP in gynecological malignancy patients (*OR* = 0.985, *P* = 0.035), aligning with findings by Li et al. (30). In cancer care, spouses often serve as primary caregivers, whose attitudes and behaviors significantly influence patients’ psychological states. According to the Stress and Coping Theory (31), individuals’ responses to stressors are closely linked to their adopted coping strategies. Dyadic coping, as an interactive approach, emphasizes collaborative efforts between partners through joint discussions, mutual support, and shared problem-solving to mitigate the adverse effects of stress. When couples employ positive dyadic coping strategies (e.g., supportive engagement, cooperative problem-solving), such collaboration reduces mutual FoP levels while enhancing resilience and adaptive capacity. These constructive interactions not only strengthen marital understanding and support but also bolster confidence in confronting disease challenges, thereby alleviating patients’ fears of progression and psychological burdens. Conversely, negative dyadic coping patterns, characterized by communication breakdowns or relational conflicts, may exacerbate psychological distress and elevate FoP severity. Thus, spousal interactions and coping styles play a pivotal role in safeguarding patients’ mental health. To effectively mitigate FoP, interventions should holistically address the needs of both patients and spouses, leveraging their dyadic relationship to develop tailored support strategies that foster emotional connection and joint coping efficacy.

#### 4.2.3 Mindset bias

The results demonstrated a significant association between mindset bias and FoP, with patients exhibiting more negative mindsets reporting higher FoP levels. These findings are consistent with Li et al. (15), who observed elevated FoP among pessimistically inclined patients compared to their optimistic counterparts. The Emotion Regulation Theory provides a framework for understanding this relationship, positing that individuals’ emotional outcomes depend on their cognitive appraisal of stressors and subsequent regulatory strategies (32). Patients with negative mindset biases frequently adopt maladaptive emotion regulation tactics, such as avoidance, suppression, or denial, which amplify FoP severity. When perceiving disease progression as uncontrollable, patients may spiral into negative emotional cycles, intensifying fear and anxiety. In contrast, positive mindset biases facilitate adaptive strategies, including cognitive reappraisal, proactive support-seeking, and problem-focused coping, which mitigate emotional distress and reduce FoP. To counteract negative mindset biases, targeted psychological interventions, such as positive reappraisal training and optimism-building exercises should be implemented. These approaches help patients reframe threats, cultivate adaptive coping skills, and disengage from detrimental emotional patterns, ultimately lowering FoP levels and enhancing disease adaptation.

#### 4.2.4 Elevated tumor markers

This study identified elevated tumor markers as a critical predictor of FoP in patients with gynecological malignancies. Tumor markers, serving as specific biological indicators of tumor presence and growth, act as a bridge between physiological and psychological states. The mechanisms underlying this association are threefold: First, direct fear induction. Elevated tumor markers are often perceived by patients as signals of cancer recurrence, metastasis, or disease progression. For instance, increased carcinoembryonic antigen (CEA) levels may directly trigger concerns about tumor advancement. Furthermore, the inherent variability of tumor marker fluctuations introduces diagnostic uncertainty, making it challenging for patients and families to interpret the clinical significance of such elevations. This ambiguity amplifies anxiety about future disease trajectories, thereby exacerbating FoP severity (31). Aligned with the Emotion Regulation Theory and Stress-Coping Model, such uncertainty activates stress responses, particularly via the hypothalamic-pituitary-adrenal (HPA) axis, leading to heightened cortisol secretion and intensified fear-anxiety cycles. Second, psychophysiological vicious cycles. Chronically elevated cortisol levels impair emotional stability and compromise immune function, fostering chronic inflammation. These physiological alterations not only degrade health status but also deepen patients’ apprehensions about prognosis, further elevating FoP. Consequently, a bidirectional relationship emerges: fear exacerbates immunosuppression, while weakened immunity reinforces disease-related anxieties, creating a self-perpetuating loop (33).Third, indirect effects of treatment adjustments. Clinically, rising tumor markers often prompt therapeutic modifications, such as intensified regimens or alternative therapies. Patients frequently interpret these changes as indicators of disease deterioration, indirectly amplifying FoP. These findings underscore the dual imperative in clinical practice: while monitoring tumor markers for biological progression, clinicians must concurrently address their psychological repercussions. Proactive psychological support and emotional counseling can mitigate anxiety triggered by biomarker fluctuations, thereby reducing FoP and enhancing quality of life.

#### 4.2.5 Symptom distress

This study revealed a significant association between FoP and symptom distress, consistent with findings by Dinkel et al. (34). During disease progression and treatment, patients commonly experience physiological symptoms such as fatigue, pain, and nausea, which frequently coexist with psychological symptoms like anxiety and fear. Their interaction collectively amplifies psychological burdens, thereby elevating FoP levels. Research indicates that emotional symptoms dominate the perioperative symptom profiles of gynecological malignancy patients (35), underscoring the critical role of psychological distress in symptom management. For instance, persistent or unpredictable symptoms may heighten anxiety, further exacerbating FoP. Symptom distress impacts psychological states via dual pathways: directly through heightened subjective appraisals of disease threat and indirectly by depleting patients’ emotional regulation resources. The Symptom Management Theory posits that patients must concurrently address physical symptoms and associated emotional responses (36). Effective symptom management thus requires not only alleviating physical discomfort but also mitigating psychological distress, particularly negative emotions. These insights advocate for integrated clinical frameworks that simultaneously monitor physiological symptoms and psychological indicators, enabling early detection of mental health risks. Furthermore, combined interventions targeting symptom relief and cognitive restructuring should be prioritized to achieve holistic care and enhance patients’ overall well-being and quality of life.

#### 4.2.6 Financial toxicity

Financial toxicity emerged as a significant predictor of FoP in gynecological malignancy patients, aligning with Li et al.’s findings (30). Its psychological impact is particularly pronounced, as financial strain and disease-related fears synergistically exacerbate psychological burdens. Patients facing economic pressures often grapple with dual fears: anxiety about discontinuing treatment due to financial constraints (potentially accelerating disease progression) and apprehension about sustaining treatment-induced economic hardships. This dilemma forces patients into difficult trade-offs between continuing treatment and managing financial strain, intensifying fears of uncertainty. Financial toxicity may also distort treatment decision-making, prompting patients to prioritize cost over efficacy by opting for suboptimal yet affordable therapies, a choice often accompanied by regret and heightened anxiety. Additionally, financial toxicity can trigger identity crises, as patients may internalize shame over their inability to afford care, eroding psychological resilience and amplifying fears of recurrence. To disrupt this vicious cycle, clinical practice should implement comprehensive support systems, including financial counseling to clarify treatment costs and reduce decision fatigue, alongside establishing peer support platforms to alleviate fear stemming from economic pressures. Such strategies may mitigate financial burdens, alleviate FoP, and ultimately improve quality of life.

Fear of Progression in cancer patients represents a complex psychological challenge shaped by multidimensional determinants spanning physiological, psychological, social, and economic domains, while being susceptible to triggering factors such as follow-up examinations, impending treatment completion, negative communication, and illness uncertainty (37). The prediction model developed in this study integrates critical predictors—including social support, dyadic coping, mindset bias, elevated tumor markers, symptom distress, and financial toxicity, to enable precise identification of high-risk FoP patients, thereby offering actionable targets for early clinical intervention. Beyond guiding personalized strategies (e.g., intensifying emotional support for patients with low social support or providing financial assistance to those experiencing severe financial toxicity), the model facilitates optimized resource allocation by prioritizing high-risk populations, thereby enhancing care efficiency. Furthermore, applying the model to assess FoP risk across treatment phases (e.g., during follow-ups or near treatment completion) and contextualizing triggers can advance whole-cycle management and multidisciplinary collaboration, comprehensively improving patients’ psychological well-being and quality of life. By leveraging this predictive tool, healthcare providers can more effectively assist cancer patients in navigating FoP, ultimately achieving the nursing goal of integrated biopsychosocial care.

### 4.3 Optimal predictive performance of the random forest model

This study compared the comprehensive performance of three models across training and testing datasets, revealing robust predictive capabilities for all models but notable performance disparities. Crucially, the Random Forest model demonstrated optimal predictive efficacy, aligning with findings by Cui et al. (38) in clinical outcome prediction using machine learning. Although logistic regression and support vector machine models exhibited stable performance with satisfactory accuracy in both datasets, the RF model consistently outperformed them. The RF model excels in handling complex medical data, offering enhanced reliability for clinical decision-making. While LR provides strong interpretability and computational efficiency, its performance may degrade under limited sample sizes or violations of linear assumptions. SVM, advantageous for high-dimensional data and complex classification problems, demonstrates robust generalization by minimizing overfitting, particularly in scenarios with small samples and high feature dimensionality. However, SVM’s sensitivity to hyperparameter tuning (e.g., kernel selection, penalty parameter *C*, and kernel coefficient *γ*) demands substantial technical expertise, and its lack of direct probability output limits intuitive applicability in probabilistic estimation tasks. In contrast, the RF algorithm, an ensemble learning method, harnesses the collective predictions of multiple decision trees to enhance overall accuracy. By aggregating diverse trees, RF mitigates overfitting risks inherent to individual trees, thereby improving prediction stability and precision. This approach imposes minimal data type restrictions, autonomously capturing feature interactions and nonlinear relationships. Furthermore, RF employs bootstrap aggregation to train trees on resampled datasets, maximizing sample utilization and refining predictive robustness (39). These attributes collectively enable RF to deliver superior and generalizable predictions in clinical settings.

## 5 Limitations

This study has several limitations. First, constrained by the research team’s limited expertise in advanced mathematics and statistics, model parameter optimization relied on conventional approaches, potentially restricting performance refinement. Second, as a single-center investigation, the absence of external validation due to time and resource limitations may compromise the generalizability of findings. Future studies should engage specialized statisticians to implement advanced parameter-tuning techniques, thereby enhancing model accuracy. Expanding sample sizes, incorporating multicenter data, and conducting prospective external validations are recommended to strengthen reliability and clinical applicability.

## 6 Implications

The high prevalence of FoP among gynecological malignancy patients underscores the clinical imperative for early identification and systematic screening. Healthcare providers should prioritize FoP assessment in routine care. Furthermore, the identified predictors, social support, dyadic coping, mindset bias, elevated tumor markers, symptom distress, and financial toxicity provide actionable targets for developing individualized precision care interventions. Tailored strategies addressing these factors may improve patients’ quality of life and long-term prognosis.

## 7 Conclusions

This study developed multiple machine learning-based prediction models for FoP in gynecological malignancy patients, with the Random Forest model exhibiting optimal performance. Critical predictive factors include social support, dyadic coping, mindset bias, elevated tumor markers, symptom distress, and financial toxicity. The integration of multiple models effectively captures the complex interplay of multidimensional predictors, providing a scientific foundation for early FoP detection and personalized intervention strategies. These findings underscore the clinical utility of combining machine learning approaches with social-ecological theory to advance precision nursing practices in psycho-oncology care.

## Data Availability

The raw data supporting the conclusions of this article will be made available by the authors, without undue reservation.
